# Perceived impacts of a school-based growth mindset programme ‘Mindset Teams’ in Scotland: a qualitative study

**DOI:** 10.1186/s12889-024-19611-6

**Published:** 2024-08-13

**Authors:** Kelly Morgan, Hayley Reed, Samantha Garay, Frank de Vocht, Simon Murphy

**Affiliations:** 1 Centre for Development, Evaluation, Complexity and Implementation in Public Health Improvement (DECIPHer), Cardiff University, Spark, Maindy Road, Cardiff, CF24 4HQ UK; 2 Centre for Public Health, Population Health Sciences, Bristol Medical School, University of Bristol, Canynge Hall, 39 Whatley Road, Bristol, BS8 2PS UK

## Abstract

**Background:**

Teachers as delivery agents within school-based mindset programmes is a potential intervention strategy for improving pupils’ outcomes. The Mindset Teams programme, utilises teachers as delivery agents, with an aim to improve learning resilience for health and attainment outcomes among schools in Scotland. This study examined the perceived impacts of the programme to develop a programme theory of change and refine an earlier co-produced logic model.

**Methods:**

Across six intervention schools, one-to-one interviews were conducted with teachers (*N* = 18) and focus groups were conducted with 23 pupils aged 8-11-years. Fourteen stakeholders involved in programme provision, secondary school delivery, funding decisions or policy-related areas also participated in a one-to-one interview. Qualitative data were analysed using thematic analysis with a combined deductive and inductive coding approach.

**Results:**

Themes were identified in relation to impacts on teachers (five themes), pupils (eight themes), and broader outcomes (two themes), with most themes discussed by teachers and wider stakeholders. Across data, findings highlighted the proposed mechanisms by which the programme was intending to produce impacts, largely through increasing teacher knowledge and facilitating a positive school environment. Themes demonstrated the breadth of perceived programme impacts, highlighting both attainment and wellbeing outcomes among pupils.

**Conclusions:**

Findings provide support for Mindset programmes focusing on the development of teachers’ mindsets, highlighting the resultant perceived impacts this can have on both teachers and pupils alike. Future research should seek to capture health and wellbeing measures alongside attainment data to fully explore programme impacts.

**Supplementary Information:**

The online version contains supplementary material available at 10.1186/s12889-024-19611-6.

## Background

The Health Promoting Schools (HPS) framework [1] encourages schools to adopt a holistic perspective with use of health educational goals. The framework emphasises the importance of supporting staff and pupil wellbeing and highlights the importance of equipping students with the transversal skills they need across the life course. Supporting students to foster a growth mindset is considered one avenue to helping students overcome daily challenges throughout life [2].

The Mindset theory (also known as implicit theories) suggests that individuals vary in their beliefs about whether human attributes (e.g., intelligence) are stable or malleable [3]. Mindset is considered important in educational settings as childhood and adolescence represent crucial periods for the development of learning abilities and skills [4–6] and both teacher and pupil mindsets have been shown to impact learning. For example, an individual’s awareness, understanding and beliefs of brain development and neuroplasticity, irrelevant of intellectual ability, have been shown to influence responses to the academic environment [3].

Recent decades have seen a surge in the number of growth mindset interventions, typically programs aiming to promote the belief that people can develop their abilities, attributes, and traits. The growing popularity of growth mindset interventions within educational settings is exemplified by several meta-analyses [7, 8]. According to the Mindset theory, pupils with a fixed mindset believe their attributes and abilities are relatively stable, or fixed, and may avoid situations in which they could struggle or fail [9]. Alternatively, a growth mindset reflects the belief that these abilities are malleable and can be developed or improved, and as such pupils with a growth mindset may seek out challenging situations [10]. The theory also motions that interventions designed to increase pupils’ growth mindsets will bring about greater academic achievement via a powerful impact of growth mindset messages upon pupils’ attainment [3].

Meta-analysis findings have demonstrated a fixed mindset is associated with mental health problems, revealing a dose relationship, with individuals who have a stronger fixed mindset more likely to report severe mental health problems [11]. Correlational research has suggested a growth mindset is associated with greater psychological wellbeing and engagement [12] and higher levels of school engagement and subjective wellbeing [13].

Previous research has focused on mindset programmes which aim to enhance pupils’ academic performance, with programmes typically focused on children aged 13-years or older. To date, findings have demonstrated mixed success, with some studies showing a positive impact on attainment scores [14, 15] and others reporting no impact [8, 16] or only a small overall effect [8]. One UK-based study focusing on younger children, aged 10-11-years across England, reported no impact on attainment scores (i.e., academic standards that children reached in three assessment areas: English, Maths and Grammar Punctuation and Spelling) [17]. Beyond educational outcomes, a recent systematic review of growth mindset interventions among school-aged children highlighted positive programme impacts on children’s physiological, cognitive, and emotional well-being, with reported improvements in growth mindset [18]. Impacts included reductions in depressive symptoms and aggressive reactions to perceived social exclusion and improved growth mindset from pre-to post-intervention for the intervention group. It should be noted however, studies included within the review were of varying research quality and predominantly focused on middle-late adolescents.

Studies indicate between 4 and 9% of the variance in pupils’ mindset is attributed to the classroom and school context [14, 19, 20]. The Mindset × Context Theory highlights the importance of considering how the environment of a psychological intervention might interplay with the intended intervention outcomes [21]. For instance, how learning opportunities within a school context might impact on results of a mindset intervention. Rege and colleagues [22] found schools offering pupils (aged 15-17-years) a choice in maths education pathways following a growth mindset intervention, had greater impacts on pupils’ academic achievements compared to pupils attending intervention schools without pathway choice. Whilst the intervention was found to be positive for both groups, the authors conclude a small yet significant Growth mindset × Context interaction was shown.

The role of the teacher in the classroom is a key contextual factor when considering school-based psychological interventions. An ecological asset within the school context, teachers have the opportunity to meaningfully influence pupils’ developments and their engagement with school [23]. There has been a recent surge in the number of programmes focusing on the development of growth mindsets among teachers. Literature suggests a teacher’s mindset can have a direct impact on a pupil’s achievement [24–26]. For example, in classrooms whereby teachers have a fixed mindset about abilities and learning, only pupils with a high ability are likely to achieve. Conversely, in classrooms where teachers adopt a growth mindset, pupils across all abilities are likely to achieve. This notion is complex and signals the interplay between the teacher and classroom environment. For instance, some studies have shown a relationship between teacher behaviours and practices and students’ perceptions of teacher mindsets [27], while others have demonstrated impacts of student perceptions of their teacher’s mindset on academic achievement (e.g., attendance and grades) [28]. These findings align with the Developmental Systems Theory (DST) which postulates that characteristics of teachers and students define how an interaction will occur and be perceived, thereby setting expectations for future interactions that influence the development of students [29]. Cross sectional studies have also demonstrated positive associations between teacher mindset and a range of outcomes, revealing greater dimensions of personal and professional wellbeing [30] and higher life satisfaction and academic self-efficacy for mastering technology [31] among teachers with a growth mindset. Considering earlier reports of high workloads, increasing stress and poor wellbeing among teachers [32–34], growth mindset interventions could offer an avenue to support teacher wellbeing.

Evidence on the effectiveness of mindset programmes targeting teachers has started to emerge. In Israel, improvements in high-school maths teachers’ growth mindset, professional well-being, and emotional teaching efficacy were found after a year-long growth mindset intervention [24]. Shoshani [35] and colleagues compared outcomes between an intervention group and control group at 9-months post intervention, with authors also reporting that improvements in teacher wellbeing were associated with increased student grades and lower levels of absenteeism. Similarly, a qualitative study in England demonstrated positive impacts of growth mindset interventions on teachers’ emotional- (e.g. greater enjoyment and decreased stress), social- (e.g. improved relationships with others) and professional wellbeing (e.g. parent-teacher communication) [36]. Recognising the influence and supporting role of teachers on pupil mindset, one UK-based growth mindset programme involved an 8-week programme of short professional development courses for teachers and workshops for pupils. While the evaluation of this study did not assess programme impacts on teachers, the randomised controlled trial revealed no impacts on pupils’ academic achievement [17, 37].

With an increasing focus on teachers as the delivery agent within growth mindset interventions, in addition to teacher impacts being a key intervention mechanism (i.e., bringing about intended impacts for teachers will lead to intended impacts on pupils), there is a need to better understand how such interventions are intended to work, and to explore intervention impacts beyond a student’s academic achievement. To date, no published studies have explored the impacts of a school-based growth mindset programme on teacher and pupil outcomes, while considering contextual moderators (e.g., school culture). Furthermore, wider studies considering impacts among younger children, aged 9-11-years, are scarce.

The aim of this paper was to qualitatively examine pupil, teacher, and key stakeholder (e.g., programme developers, training deliverers etc.) perceptions of the theory of change of the Mindset Teams programme. Findings have been used to inform the refinement of an intervention logic model (see Additional file 1) co-produced prior to the study with intervention developers, deliverers, and the research team.

## Methods

### Mindset teams intervention programme

The Mindset Teams programme is currently delivered throughout primary and secondary schools across Scotland, United Kingdom, having first been introduced in 2018 [38]. The programme is delivered over a 12-month period, with schoolteachers (typically 1–3 members of staff, a Mindset Leader and two Mindset Champions) taking part in a 6-month online training course followed by a further 6-month period for the practical application of course learning within the classroom or across the school. The programme ultimately aims to promote a growth mindset culture within schools, with the online course designed to improve staff knowledge and confidence around mindsets and around change management in schools. The programme has been described in greater detail elsewhere (paper under review).

### Study design

This was a qualitative study using one-to-one interviews with stakeholders and teachers and focus groups with pupils aged 9-11-years. The Consolidated criteria for Reporting Qualitative research checklist (COREQ) [39] was consulted for reporting the study.

### Participants and methods

#### Wider stakeholders

Stakeholders (*N* = 48) involved in (i) programme development, training or monitoring (ii) programme delivery in secondary schools, (iii) programme funding decisions or (iv) the policy arena, were invited to participate in a one-to-one semi-structured interview. Using convenience sampling, a study invite letter and information sheet were sent to a list of programme contacts held by Winning Scotland (programme provider) and policy contacts sourced with publicly available information. Of the 48 stakeholders invited, 14 took part in an interview, six declined and 24 did not respond. Participants included four programme providers, four secondary school representatives (three teachers and a Head teacher), two local authority education officers and four national stakeholders. During interviews, a topic guide was used to explore four key programme areas: uptake; implementation; sustainability and perceived impacts on pupils and teachers. This paper focuses on the perceived programme impacts.

#### Schools and teachers

Primary schools who had previously enrolled in the Mindset Teams programme were emailed an invitation to participate in the study. Six schools were recruited to the study, all having commenced the programme since 2021. Schools spanned four local authorities, with three schools residing in one authority. Based on Scottish Index of Multiple Deprivation (SIMD) data [40], two schools (IDs 002 and 006) were located in an area of high deprivation, three schools within an area of medium deprivation (001, 004 and 005) and one school a low deprivation areas (003).

At each school, up to four teachers involved in the programme (including the Head Teacher, a Mindset Leader and two Mindset Champions) were invited to take part in a one-to-one semi-structured interview. A total of 18 school staff took part, including six head teachers and 12 teachers. Teacher interviews lasted between 25 and 50 min. In addition to the questions covered in the stakeholder topic guide, teachers were asked specific questions regarding the programme and school setting (e.g., role of the wider school-community in supporting growth mindset).

#### Pupils

Within each participating school, pupils aged 8-11-years (i.e., P5-P7 / Year 4–6) were invited to take part in an online focus group. As part of the pupil recruitment process, schools were asked to give each pupil a child-friendly study information sheet (co-created with children in our Public Involvement school) and send study information sheets and consent forms to parents/carers using the school’s existing systems for communicating with parents. This ensured parents/carers had the opportunity to speak with their child about the study and opt their child into the study should they wish to do so. Of the children with parent/carer consent, teachers were asked to nominate and invite six-to-eight children to participate, with an even distribution of both sexes. The teacher was provided with a script to explain the nature of the focus group and the voluntary nature of participating. If a child declined to participate, the teacher was asked to invite an additional child. A topic guide was developed to explore pupils’ experiences and understanding of the programme, the influence of others on growth mindset and perceptions of programme impacts. Focus groups were designed to include the use of creative and visual methods (e.g., drawing activities to depict understanding of growth mindset, hands-up activities to indicate agreement with wider group ideas), which are increasingly used amongst children and young people to support and encourage discussion [41]. In total, four focus groups were conducted with 23 children (aged 8–11 years) across four schools, with discussions lasting on average 29 min (range 25–34 min).

### Research conduct and study team

All data collection took place between September 2021 and July 2022. Interviews were conducted via telephone or virtually using Teams depending on the discretion of the participant. All focus groups took place remotely via Teams with a schoolteacher accompanying children throughout the discussions. Working with a public involvement school in Scotland, two remote sessions with teachers (*N* = 3) and two sessions with pupils aged 9-11-years (*N* = 5) tested and refined school recruitment methods and data collection tools. All adult participants provided written and/or audio consent for participation, audio recording and use of anonymised quotations. Pupils provided written assent prior to the focus group commencing.

Qualitative data were collected and analysed by the lead author (KM) and study manager (HR), with analyses supported by SG. KM and HR have undertaken formal training with qualitative research and KM mentored SG throughout analyses. All the research team members were women (for reporting purposes of item 3 on COREQ checklist [39]). No prior relationship between the research team and school participants was established.

### Analysis

Data were transcribed verbatim and analysed using NVivo 12 (QSR International Pty Ltd) software [42]. Transcripts were checked for accuracy and to ensure anonymisation. The inductive thematic approach of Braun and Clarke [43] was utilised for data analysis, beginning with an initial review of transcripts. A conceptual framework for each dataset was produced that included initial themes and sub-themes. As part of this process the research team read and open coded a small number of transcripts in each set before meeting to discuss and amend the framework to best fit the data, study objectives and research questions. Upon agreement of initial codes and frameworks, a systematic approach to data management was adopted, with all transcripts coded into the framework. Once all transcripts were coded the team had a detailed and accessible overview of the data populating each theme and sub-theme for every respondent. Participants were not provided with transcripts for comment or correction however, overall research findings were later shared.

## Results

Table 1 displays the characteristics of participant groups.

**Table 1:** Overview of school participants where focus groups and interviews were conducted

School ID	Participant/Group ID	Date of data collection	Number of participants	Number of females	Number of Males
001	001 HT	Jun-22	1	1	
	001 ML1	Apr-22	1	1	
	001 MC1	Jun-22	1	1	
	001 MC2	Jun-22	1	1	
	001 FG1	Oct-22	4	2	2
	001 FG2	Oct-22	7	4	3
002	002 HT	Jul-22	1	1	
	002 ML1	Jun-22	1	1	
	002 MC1	May-22	1	1	
	002 MC2	Jun-22	1	1	
003	003 HT	Jun-22	1		1
	003 ML1	Jun-22	1	1	
	003 MC1	Jun-22	1	1	
	003 MC2	Jun-22	1	1	
	003 FG	Oct-22	5	4	1
004	004 HT	May-22	1	1	
005	005 HT	Jun-22	1	1	
	005 MC1	Jun-22	1	1	
	005 MC2	May-22	1	1	
	005 FG	Nov-22	3	2	1
006	006 MC1	Jun-22	1	1	
	006 MC2	Jun-22	1	1	

Results relating to perceived programme impacts on teachers are first discussed, followed by impacts on pupils and lastly wider impacts are highlighted. Findings have been used to further refine the logic model co-produced in 2020. Exemplar quotations illustrate responses across themes (participants denoted as: HT = Headteacher, MC = Mindset Champion, ML = Mindset Leader, FG = pupil focus group, WS = Wider stakeholder, with accompanying numbers showing a participant ID or group number).

**Fig. 1:**
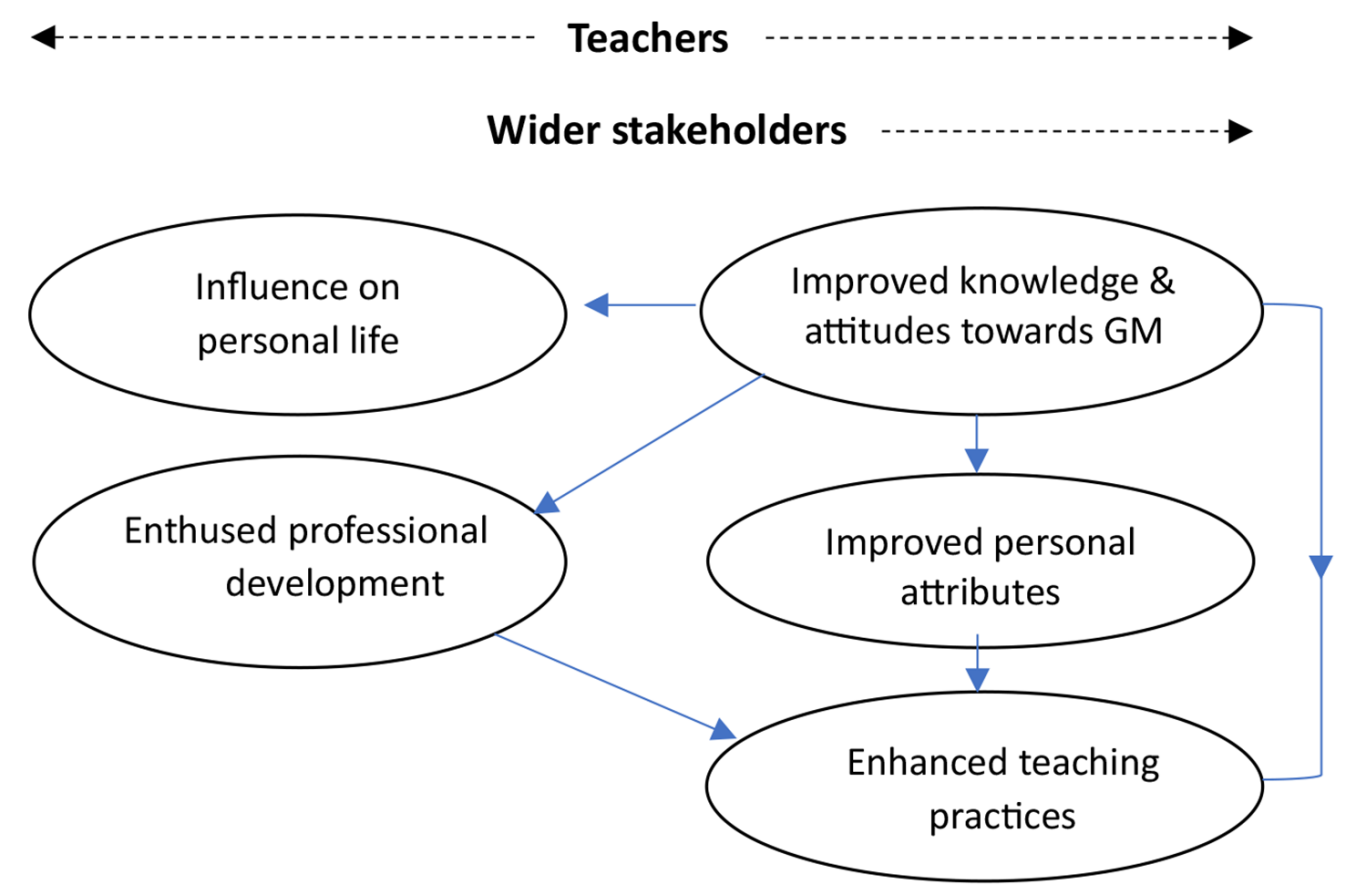
Perceived programme impacts on teachers as identified by teachers and/or wider stakeholders

Five themes concerning teacher impacts were identified across teacher and wider stakeholder data; improved knowledge and attitude towards a growth mindset, development of personal attributes, enhanced teaching practice, enthused professional development and influences on personal life. The final two themes were identified in teacher data only. Figure 1 depicts each theme and data source, highlighting areas of similarity and differentiation across the two participant groups.

**Fig. 2:**
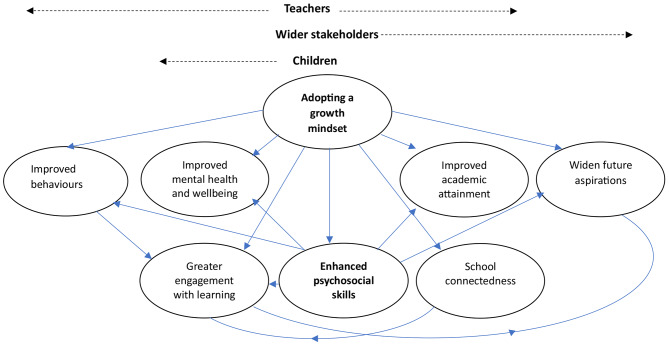
Perceived programme impacts on children, identified by teachers, children and/or wider stakeholders (bold indicates all groups identified impact e.g., adopting a growth mindset was identified by all three participant groups). Arrows indicate the perceived direction of impact (e.g., enhanced psychosocial skills support greater engagement with learning)

Eight themes were identified in relation to programme impacts on children; adopting a growth mindset, enhanced psycho-social skills, improved behaviours, school connectedness, engagement with learning, academic attainment, wider aspirations and improved mental health and wellbeing. Two of these themes were discussed by all three groups (adopting a growth mindset and enhanced psychosocial skills). Figure 2 displays all eight themes with an indication of which groups identified common or different themes.

### Impacts on teachers – data from teachers and wider stakeholders

#### Theme 1: Staff knowledge and attitudes towards growth mindset

Teachers and wider stakeholders recognised the impact of the programme on teachers’ understanding of growth mindset, with improved knowledge perceived as a key mechanism in achieving wider outcomes. Some teachers described instances where they had adopted growth mindset aspects within their teaching, however, undertaking the course had made them recognise how limited their earlier understanding was.

*I do feel especially me at the beginning of the project*,* I felt I was a teacher that would go around saying*,* ‘Oh*,* yeah*,* I already do growth mindset’*,* but I do think it’s a term that is thrown around*,* it’s become a buzzword*,* ‘Oh yes*,* we’ve all got growth mindsets*,*’ but looking back now*,* I didn’t*,* and I think a lot of other teachers will be the same. (001 MC2)*

Other teachers talked about how the course led them to recognise their own fixed mindset and how gaining further knowledge had enable them to understand and embrace a growth mindset. This recognition was also described among wider stakeholders, with one stakeholder highlighting an even greater perceived impact on teachers with fixed mindsets and subsequently pupils.

*I think initially there is always that little bit of shock*,* sometimes a few demons that you didn’t realise existed in your thinking and I think those reluctant teachers are often aware that they don’t have a growth mindset and so they are the hardest people to reach*,* but interestingly if you can reach them*,* they are often the best converts as they are passionate about turning things around for children. (WSI 11)*

In two instances, teachers described situations whereby they faced high levels of scepticism among other staff members and through sharing their new knowledge, this affirmed their own attitudes and learning towards growth mindset. Throughout wider stakeholder interviews, the impact of the programme on staff attitudes was recognised as a key mechanism to supporting pupil learning.

#### Theme 2: personal qualities

This theme comprised two sub-themes; sense of enthusiasm and improved confidence, patience and empowerment.

#### Sense of enthusiasm

Teachers described a greater sense of enthusiasm, in which they were excited and passionate about growth mindset as they could see the applied learning making a difference within the classroom. Others reported improved job satisfaction which was associated with the greater excitement around learning and improved personal mental health. However, a lot of staff had not previously considered the impacts of the programme on their own wellbeing.

*… for me getting that excitement again about my own learning has made me enjoy my job again more which has definitely impacted on my mental health*,* in a round about way I suppose… I’ve always loved my job … but recently I’d started to feel like a bit like okay*,* well this is me in my job now and like*,* I don’t always love it*,* but I think getting excited about my own learning again*,* I started focussing on new things after doing this course and thinking about how I could grow my own brain I think has definitely helped me. (003 MC2)*

#### Improved confidence, patience and empowerment

There were various accounts of how the programme had led to improved confidence and greater empowerment among teachers. Teachers described how the course had enabled them to be creative in their teaching approaches and be confident when using new vocabulary with the children to facilitate conversations around growth mindset. Wider stakeholders at the local and national level also recognised the impact of the course on teacher confidence.

*It’s all about that excellence stance*,* that practitioners are confident and inspired and*,* therefore*,* they provide excellent learning and teaching opportunities for the learners. (WSI 8)*

Two staff members highlighted improved patience because of the programme. One teacher described that due to children being more engaged in learning, this had created a more pleasant school environment for teaching which resultantly supported them to adopt a more patient teaching approach. Another teacher relayed how their new knowledge and appreciation of learning states had encouraged them to allow more time for pupils to work through activities.

*I think as a teacher it teaches you to be patient don’t jump in there*,* let them. It’s not floundering but let them find out for themselves a little bit because otherwise if you’re always saying ‘it’s alright we’ll fix that or you keep doing it’ they’re never going to learn…’ (005 MC1)*.

#### Theme 3: application of growth mindset to personal life

This theme was informed only by teacher data, with discussions highlighting how an improved understanding and attitude towards a growth mindset had informed situations beyond the school environment. Teachers described scenarios where they had applied their own learning to situations with family, friends and home life.

*I learned about you know*,* how I deal with people*,* I learned about how I support people in the workplace*,* I learned about how I speak to my own children*,* you know*,* it had an impact … I think it’s so much more further reaching than just the classroom*,* you know*,* it has such an impact on me as a practitioner but also me as a human being*,* you know*,* so the influence has been much more wider reaching than just school life – it’s family life*,* it’s home life*,* it’s you know*,* my wider circle of friends as well. (003 ML1)*

On the contrary, some teachers had not considered the impacts on teachers’ personal lives while one headteacher felt it was too early to determine any impacts beyond those observed among the pupils.

*I can’t say that I’ve seen a sort of impact across the staff in general just now because it’s still in the kind of early stages of rolling it out. Certainly in [Teacher named] class I would say with her*,* it’s very much a case of she feels that she’s kind of getting more out of her lessons. The children are much more engaged now and the learning and she feels that she’s getting through a kind of period of work with them.’ (002 HT)*.

#### Theme 4: enhanced teaching practices

Among wider stakeholders there was a perception that teachers attending the programme would shift their practice from a more traditional ‘command and control’ approach to teaching in a more facilitatory manner, whereby children become involved in constructing knowledge. Similarly, teachers spoke about how the programme had impacted their teaching practice, with many directly applying the learning from the programme to their teaching. It was highlighted that higher standards of teaching had been brought about through improved communication with the children and the improvements to the teacher’s ability to better identify the needs of the students.

*Our teaching resources and tasks that we’re giving are to a much higher standard because we are thinking about what challenges are going to promote the higher order thinking skills*,* what challenges are going to promote communication … We’re teaching different methods and we’re exploring different ways with the children and celebrating that it’s okay that you’re doing it differently to somebody else. (002 MC1)*

#### Theme 5: enthused professional development

Several teachers spoke about the impacts of the programme on their professional development, with a renewed sense of enthusiasm for their own educational journey. For some, this enthusiasm had led to the enrolment in additional mindset courses to further learning in addition to wider professional development. One teacher spoke about being inspired to enrol in further postgraduate education.

*I’ve never listened to so many podcasts about growth mindset or looked at so many projects that are connected with it*,* past finishing the course. Each day when I drive to work I listen to a little bit more information about it. (006 MC1)*

### Impacts on pupils – data from teachers, wider stakeholders and pupils

#### Theme 1: adopting a growth mindset

There was a strong recognition of the programme’s impact on children adopting a growth mindset. Specifically, wider stakeholders and teachers described the role of the programme in creating more independent learners through instilling skills to support learning resilience. Teachers further emphasised that growth mindsets were portrayed across different areas of the curriculum, not just specific lessons where the growth mindset programme had been specifically implemented, sharing explicit examples across the school curriculum. Among three focus group discussions, children talked about how their growth mindset learning had encouraged them to keep trying when they faced difficult classroom activities as opposed to giving up easily, which they would have done previously.

*It could help you in maths*,* if there was a really hard calculation. If you didn’t know growth mindset and there was a really hard calculation you’d probably and if you said you can’t do it*,* you’d probably just give up. Or and if you knew what growth mindset is and gave it a go you might*,* you could get it right. (003 FG)*

#### Theme 2: enhanced psycho-social skills

This theme was largely discussed by teachers and pupils, with three underlying sub-themes, improved communication skills, increased confidence, and improved social skills. Wider stakeholders did however discuss the role of the programme in increasing children’s confidence.

#### Improved communication skills

Pupils described how the programme had improved their ability to communicate effectively with both peers and teachers, and how this had in turn increased their confidence to engage within the classroom. Teachers also recognised enhanced communication skills, with children widening their vocabulary and framing their learning in more positive terms.

*I find that my friends are really helpful. Because I didn’t really understand a task or I didn’t really understand because sometimes I was off (absent) and my friends explained it to me. (003 FG)*

#### Increased confidence

Discussions among teachers, wider stakeholders and pupils relayed how the programme impacted on children’s confidence. Stakeholders described how the programme empowered children with new skills which supports them to feel less intimidated by new situations and challenges. One pupil mentioned their new ability to share ideas and ask questions as a result of increased confidence through the programme.

*Most of the time I feel confident. Because … before you can get frustrated because you can’t really do it but then once you get it*,* you’re like*,* ‘oh*,* I can do it’. (001 P5)*

#### Improved social skills

Teachers spoke about greater class cohesiveness as a result of the programme. Witnessing pupils adopt a growth mindset and supporting one another in class, were both identified as factors adding to a positive class atmosphere. Across three schools, teachers also emphasised how pupils’ improved social skills had been transferred beyond the classroom, with the building of new and existing friendships and better use of conflict resolution in the playground. One teacher described the immediate changes to the class dynamic, with greater levels of peer support and calm towards one another.

*So my class that couldn’t sit together couldn’t look at one another*,* couldn’t travel out of the school building together*,* to be in a room supporting one another with open body language is a huge change. (006 MC1)*

#### Theme 3: improved behaviours

Most teachers highlighted the programme’s impact on children’s improved behaviours. Teachers described how children would often act out in class and become disruptive in a reaction to them feeling challenged with a task. With use of the growth mindset programme and better communication skills, teachers acknowledged that it was now less of a battle with children’s behaviours, and that led to pupils being were more engaged in learning.

*… at the start of the year*,* sort of classes maybe not being engaged*,* not being on task*,* kind of getting distracted and for us*,* we were called quite a lot to support pupil behaviour and a lot of times when you were speaking to children it was very much like ‘I don’t know what I’m doing*,* I don’t understand it*,* what’s the point in me learning*,* what do I need it for’. Whereas I have to say … we’re not called for because it’s very much a case of the whole mindset has shifted … (002 HT)*

#### Theme 4: School connectedness

Wider stakeholders, particularly programme providers and secondary school teachers highlighted how the programme can impact on children’s sense of belonging and connectedness to schools. Specifically, stakeholders described how increased school connectedness was likely attributed to the new language style teachers were using to ensure children felt in a safe, supportive environment. Often this was perceived as most beneficial for those children who had low engagement or low school attendance rates.

*We had six 15-year-olds who were disengaged from school*,* they were behaviour issue kids*,* they were non-attenders*,* and they were at the point of leaving school with no qualifications and no point of destination. And through the course it was the emotional side of working with the kids*,* so they had an impact on the feeling of belonging*,* of being part of the school*,* that people cared to open up to deal with their emotions*,* all through the change of language on how we could help them be in school every day. (WSI 3)*

#### Theme 5: Engagement with learning

Most teachers spoke of how the programme facilitated student’s engagement in learning. For some, this had been achieved by giving children more ownership over their learning, with children now able to identify their own challenges and communicate to the teacher whether they need more or less stimulus within classroom activities. For others, teachers described how the programme had created a greater sense of enjoyment for learning among children. One teacher also described how their school was aiming to improve pupil attendance by supporting growth mindsets among pupils and reducing negative learning experiences. Children also recognised the influence of the programme on learning engagement, as children across two focus groups mentioned the ability to focus better on their work when they adopted a growth mindset.

*I would say that’s the best impact of the project in my class*,* they were enjoying learning about it*,* and I felt I was getting more from them. (001 MC2)*

#### Theme 6: academic attainment

Stakeholders at the national level motioned how the development of growth mindsets will in time lead to positive impacts on educational attainment, a longer-term impact also anticipated by teachers. Across two schools however, teachers spoke of the observed programme impacts on children’s academic learning outcomes, describing specific examples of improved literacy scores within their schools.

*Writing lessons were one of the hardest lessons*,* there were always problems*,* big*,* big*,* problems where the lesson would be completely interrupted with their behaviour*,* and most children didn’t get the chance to write because it seemed to cause quite a lot of stress. So the writing results have improved as well*,* I think we’re talking about 67% I’m sure it is*,* the writing results have gone up just over the term. (006 MC1)*

While the impacts of the programme on academic progress were described, most teachers stressed how the programme was creating benefits far beyond academic achievement. One head teacher spoke about the need to take a holistic lens to assessment, encompassing achievements in life skills and academic achievements. The inability to distinguish impacts caused by Mindset Teams and wider programmes or influences was also acknowledged by many.

#### Theme 7: wider aspirations

Wider stakeholders at the national level spoke about the programme impact on raising children’s future aspirations. Within discussions, stakeholders described that through greater engagement in learning and increased self-confidence, children are then able to recognise that they can succeed and this will in turn influence what they aspire to do in the future. While not explicitly mentioned within teacher discussions, one national stakeholder noted how this impact was a key driver among schools and areas involved the programme.

*That*,* for most people I’ve encountered*,* whether it be at school level or at central level*,* is a feeling that they’re always looking for something that’s likely to raise attainment and there’s been an acknowledgement that growth mindset often has a positive impact. (WSI 2)*

#### Theme 8: improved mental health and wellbeing

The impact of the programme on children’s mental health and wellbeing was highlighted by teachers across all schools, with further recognition within one pupil focus group. Teachers described how the children were happier since the programme had been implemented, and how this was linked to the wider impacts on children’s communication skills, increased confidence, and a recognised need to place less pressure on themselves within tasks. In one school, teachers described how the programme supported children’s social and emotional needs, with a school survey demonstrating improvements in personal attributes such as positivity, motivation, and resilience. When describing improvements in emotional resilience, one teacher noted that whilst you can see these improvements in the classroom they are hard to evaluate.

*So yeah*,* I think they’re happier*,* I think they’re more relaxed*,* I think they’re more open and willing to learn new things*,* and that actually learning new things is good fun. So*,* that’s the effect it has on their wellbeing (001 ML)*

One pupil described how learning about growth mindset had helped to support their understanding of how the brain works while in different mindset states and in turn that helped to support their mental health.

*… it’s helped with our brain and our mental health. Because we were learning about our body and [Teacher named] was focused on our brain and how the neurons are always firing but if we had a fixed mindset the neurons are tired and they can’t fire that well and if you had growth mindset all the neurons are going to be firing and building bridges*,* so we can walk across them. (003 FG)*

**Broader impacts of the programme*– data from teachers***,*** wider stakeholders and pupils***.

Two further themes demonstrated impacts across the wider context; building a positive school culture and the transferability of new skills to the home setting.

#### Theme 1: building a positive school culture

This theme was underpinned by data from wider stakeholders and teachers, and comprised three sub-themes, sharing of growth mindset practice, engaging parents, and strengthening school leadership.

#### Sharing a growth mindset practice

Wider stakeholders and teachers acknowledged the role of the programme in supporting a cultural shift to embed growth mindset across the whole school. Teachers spoke about the programme creating a positive upbeat culture at school, as staff shared their enthusiasm with other staff members and teachers approached learning and problem solving with a positive nurturing attitude. This was also shown through the sharing of practices and knowledge among teaching teams.

*…there’s something quite powerful about the learning being shared with the rest of the staff which we’ve started already to do. (003 HT)*

#### Greater connections with parents and caregivers

Stakeholders at the national and local level highlighted how the programme can increase links between parent, caregivers, and schools, whilst some teachers further acknowledged the wider need to support the development of growth mindsets among parents. One stakeholder highlighted how the programme had enhanced parent’s and caregiver’s appreciation of the school’s approach with children in creating a more welcoming and positive environment. This was particularly evident in areas where schools reported low levels of pupil engagement.

*What I found that was parents loved it*,* they loved the positivity of it. They loved the thought that their children were coming into school and hearing this positivity from the teachers and particularly*,* when we were saying to them this is for everything*,* even if your child is late*,* we aren’t interested in saying where have you been*,* we are saying we are so delighted you have made in today*,* wonderful*,* come sit down and join in*,* this is what we have been doing and saying to people. This has been a whole culture shift. (WSI 11)*

#### Strengthening school leadership

Impacts of the programme on school leadership were discussed among teachers across two schools. One teacher emphasised a greater appreciation for their role in the leadership of change, with a greater understanding of how to lead change while accounting for differing levels of readiness for change among colleagues. Similarly, one head teacher highlighted how the programme had focused their leadership efforts and the management of school level strategies.

*…the actual leadership bit of the course I found really useful and it has directed my school improvement plans over the last couple of years as well and it’s [senior leadership] become much more focused and much more manageable… (005 HT)*

#### Theme 2: transferability of skills

Wider stakeholders, teachers and children all highlighted the transferability of the programme impacts beyond the classroom setting. Many stakeholders relayed the potential for programme impacts to ripple across wider areas of life (e.g., sport, non-educational settings etc.), particularly with use of self-reflection and adopting the learnt skills to support a growth mindset. Several teachers described the positive comments they had received from parents, with shared examples of growth mindset learning being applied at home. Within one example, a parent had relayed how their child had ‘used a kind of growth mindset language to support them in the home’ when they were feeling stressed. Children also talked about examples of applying their growth mindset beyond the school setting, accounting scenarios at a music lessons and physical activity.

*On my birthday I got a new bike*,* and the brakes weren’t that good. So I thought whenever I was going to push the brakes*,* I thought I was going to fall off. So I think growth mindset helped me a bit there. (003 FG)*

## Discussion

To date limited research has explored the perceived impact of a school-based growth mindset programme on teachers and pupils, with consideration for the school context. This study explored perspectives among pupils, teachers and wider stakeholders who have either implemented or been involved in the development, delivery, funding of the programme or policy-related arena.

Findings of the present study demonstrate the mechanisms and broad ranging outcomes through which the Mindset Teams programme is perceived to impact teachers and children, supporting the Mindset x Context theory [21]. This study adds evidence to the growing literature regarding the importance of focusing on the development of teachers’ mindsets and the resultant impacts this can have on both teachers and pupils alike. Teachers and wider stakeholders however, recognised perceived impacts, especially on attainment outcomes, were likely to emerge over time, and acknowledged the difficulty in formally measuring impacts which had been observed in the classroom.

Many of the perceived programme impacts on teachers were underpinned by the accounts of improved knowledge and attitudes towards a growth mindset. This impact was demonstrated through our earlier quantitative analyses of teacher pre- and post-training survey data, revealing improved teacher outcomes following the 6-month online aspect of the Mindset Teams training course (paper under review). Wider impacts on improved personal attributes and teaching practices concur with recent findings. For example, Shoshani [35] found enthused professional development and greater mental wellbeing among teachers following a growth mindset intervention. Zilka and colleagues [44] found teachers with a growth mindset were more likely to experience higher levels of teaching practice and less burnout, a relationship which was explained by greater levels of teacher flow and critically reflective behaviour.

These findings are particularly pertinent considering the widespread concerns over teacher workloads and negative impacts on mental health, wellbeing, and retention [32–34]. In 2021, a national survey of 3354 teachers across the United Kingdom revealed 77% of teachers had experienced poor mental health due to work, 72% felt stressed and 54% had considered leaving their careers [45]. Enhancing teacher wellbeing has been identified as a priority to support teacher retention and thus school success [32, 46]. The present study highlights a programme ultimately aiming to impact the mindset culture of a school can also provide several benefits for both teachers and pupils. This should be an important consideration for future schools weighing up workload requirements and benefits to engaging in the Mindset Teams programme.

An array of impacts on children were highlighted across data, and these were perceived by all participant groups to be underpinned by the adoption of growth mindsets. Historically, growth mindset programmes have been used as a vehicle to support children’s academic achievement, and the present findings, along with earlier studies [14, 15], demonstrate plausible mechanisms for supporting this outcome. Wider literature has demonstrated children feeling confident in their own ability and having a sense of social belonging are important factors that support a child’s academic achievement [47, 48], impacts which were described as part of the Mindset Teams programme. Our recent analyses of routine school-level attainment data showed moderate positive associations between programme delivery and pupil writing scores, with little evidence of differential impacts in disadvantages groups (paper under review). Taken together, our study findings show promise of programme impact on attainment and wider outcomes, with several potential mechanisms described.

Present findings demonstrate the mechanisms by which a growth mindset culture can be developed within a school, with the sharing of knowledge and practice, development of community connections and strengthened leadership, identified as key ingredients. Cultivating a growth mindset culture across a school can help to embed the values, behaviours and practices of growth mindset among teachers and pupils alike. This culture would align with the HPS framework by supporting resilience and personal growth throughout the educational setting, and the implementation of a curriculum which integrates health and education using both inclusive and tailored approaches [49].

While schools are benchmarked on pupil attainment scores, there is a global movement to make every school a health-promoting school [1] and a shared ambition across Scotland to protect and nurture metal health from a young age, both within and outside of educational settings [50]. Current findings showcase perceived programme impacts beyond the school setting, with numerous accounts of improvements in pupil mental health and wellbeing, and acknowledgement for how the programme can support children’s future endeavours. These outcomes were often attributed to the development of growth mindsets and enhanced psychosocial skills, both of which, were identified as transferable across contexts and the different facets of children’s lives. Future studies should endeavour to collect quantitative outcomes on pupil health and wellbeing alongside educational data.

## Strengths and limitations

There are several strengths to this study, including the involvement of three different groups of participants across interviews and focus groups, which provided a broad range of perspectives through in-depth exploration. The inclusion of schools already implementing the Mindset Teams programme also enabled pupils and teachers to share their lived accounts and experiences. The researchers conducting the study had no prior involvement with the programme, minimising bias in reporting. Limitations of the study include the inability to account for wider school programmes or initiatives taking place concurrently which could have also had similar impacts on teachers and pupils. Considering the sample size, generalisation of findings should be done with caution as it is possible that data saturation has not been reached, and as such, some of the derived themes might not be fully developed in terms of density and variation. We also cannot rule out sampling and/or social desirability bias of participating schools, pupils and wider stakeholders who agreed to participate in the study, therefore this is important in interpreting the study findings.

## Conclusion

This study adds important findings to the literature base surrounding school-based growth mindset programmes. Findings show a multitude of perceived positive impacts of the Mindset Teams programme on teachers and pupils. Importantly, impacts were shown to span areas of school-life and health, with new skills and traits being transferred outside of the school-setting. Based on these findings, schools can better understand how implementing the Mindset Teams programme can help achieve goals within school improvement plans and ultimately contribute towards pupil attainment and wellbeing.

### Electronic supplementary material

Below is the link to the electronic supplementary material.

Supplementary Material 1

## Data Availability

The datasets generated during the current study are not currently publicly available due to wider publication plans but are available from the corresponding author on reasonable request.
