# Supplementary material for: Perceived impacts of a school-based growth mindset programme ‘Mindset Teams’ in Scotland: a qualitative study
Source: BMC Public Health. 2024 Aug 13;24:2199. doi: 10.1186/s12889-024-19611-6 (PMC11321148; doi:10.1186/s12889-024-19611-6)
Supplement: Supplementary file 1 — Supplementary Material 1 [file 12889_2024_19611_MOESM1_ESM.docx]

**Long term**

**(3yr plus)**

- Narrowing of the poverty related attainment gap
- Sustained growth mindset culture within education and school community

Trained teachers have increased knowledge of growth mindset research, principles and practices

Mindset leader to help support the project and ensure alignment with school and Scottish Attainment Challenge strategic aims.

- Online discussions
- Summative assessments

Winning Scotland: All young people in Scotland to be ambitious, versatile & resilient

Mindset champion to design and implement the project

- Online discussions
- Summative assessments

Ongoing support mechanisms

**THE INTERVENTION**

**MECHANISMS OF CHANGE**

**OUTCOMES**

Pupils receive growth mindset practices

Mindset teams develop and implement growth mindset strategies within schools.

Creation of a vibrant active learning community

Mindset teams develop a growth mindset culture within school

Trained teachers engage and exchange ideas and approaches with other teaching professionals across Scotland

Mindset in curriculum

Local Authority strategic alignment and funding in line with Local Authority improvement plan

**TEACHERS**

- Greater engagement with further training/ education

Positive school culture

**TEACHERS**

- Improved beliefs and attitudes towards pupil mindset
- Improved awareness of growth mindset practice
- Improved teaching practices
- Improved wellbeing
- Increased job satisfaction

**Short term**

**(9m - 18m)**

**PUPILS**

- Improved attainment
- Enhanced pupil psychosocial skills
- Adoption of growth mindsets
- Improved peer relationships
- Improved resilience for learning
- Improved behaviours
- Increased school connectedness

**Medium term (18m – 3yr)**

**PUPILS**

- Growth mindset embedded within school improvement plans
- Improved pupil mental wellbeing
- Improved pupil resilience
- Increased pupil confidence to pursue and fulfil their aspirations
- Improved pupil attainment (reading, numeracy and writing)

**External context and school characteristics**

Programme is delivered in areas of high deprivation (i.e. ‘Attainment Challenge’ areas in receipt of Government funding) and setting and characteristics of school (i.e. Primary and Secondary schools, different school size and levels of affluence) and the year the programme is introduced (i.e. from 2017 onwards)
